# Exploring Disproportionate Mental Health Act Detention Among Black Adult Men: A Thematic Review

**DOI:** 10.1192/bjo.2026.11543

**Published:** 2026-06-30

**Authors:** Henry Browning, Rashed Khalid, Jordan Williams, Eimen Javed, Eikay Htun

**Affiliations:** 1 Leeds and York Partnership NHS Foundation Trust, Leeds, United Kingdom; 2 Tees Esk and Wear Valley NHS Trust, Harrogate, United Kingdom; 3 South West Yorkshire NHS Trust, Wakefield, United Kingdom

## Abstract

**Aims:**

There is clear evidence that Black men are disproportionately detained under the Mental Health Act (MHA). Our aim was to explore and characterise the clinical, social, organisational and structural factors contributing to this phenomenon, by examining the pathways to compulsory admission among Black adult males, and to generate hypotheses to inform future research and service improvement.

**Methods:**

We completed a thematic analysis of Structured Judgment Reviews (SJRs) of a consecutive series of Black adult male patients detained under the MHA within a single NHS mental health trust. The MHA team provided ten consecutive cases organised by date of detention; two cases were excluded due to being minors, leaving eight cases. The reviews were completed by trained clinicians using NHS SJR methodology, to apply structured judgment and qualitative narrative across phases of care. We identified themes relating to routes to detention, (dis)engagement with services, quality of care and social context.

**Results:**

Most patients had psychotic diagnoses (schizophrenia or BPAD), with histories of multiple detentions. Patient-related themes included: disengagement from CMHT services, poor medication adherence, and substance misuse. Several patients experienced significant social adversity, including homelessness, socioeconomic deprivation, exploitation and limited support networks. Migration-related stressors, trauma and language barriers were also evident.

Regarding organisational factors, inpatient care was generally timely and of adequate or good quality, with appropriate multidisciplinary involvement. Transitions from inpatient to community care emerged as a consistent point of vulnerability. Communication difficulties between services, missed opportunities for assertive follow-up, inconsistent provision of interpreters, and delays in initiating substance misuse interventions were recurrent. In several cases, rapid relapse occurred within months of discharge, leading to repeated crisis presentations and subsequent detention.

**Conclusion:**

Our analysis of the themes suggests that compulsory admission under the MHA disproportionately affect Black males due to cumulative interactions of clinical complexity, social deprivation and system-level gaps in care continuity, rather than care quality alone. Although our findings cannot establish generalisability or causation, they are supportive for the hypothesis that earlier, more tailored and integrated community care may reduce detention for Black men. Future work could practice on larger samples and lived-experience perspective to inform culturally responsive service models and ongoing MHA reform. The Trust’s Quality Committee welcomed the report, endorsed further exploration of the key theme, and agreed the recommendations would be progressed through relevant governance committees.

